# Transcriptome Analysis and Metabolic Profiling Reveal the Key Regulatory Pathways in Drought Stress Responses and Recovery in Tomatoes

**DOI:** 10.3390/ijms25042187

**Published:** 2024-02-11

**Authors:** Jinshuai Shu, Lili Zhang, Guiming Liu, Xiaoxuan Wang, Fuzhong Liu, Ying Zhang, Yuhui Chen

**Affiliations:** 1 State Key Laboratory of Vegetable Biobreeding, Institute of Vegetables and Flowers, Chinese Academy of Agricultural Sciences, Key Laboratory of Biology and Genetic Improvement of Horticultural Crops, Ministry of Agriculture, 12 Zhongguancun Nandajie Street, Beijing 100081, China; wangxiaoxuan@caas.cn (X.W.); liufuzhong@caas.cn (F.L.); zhangying02@caas.cn (Y.Z.); chenyuhui@caas.cn (Y.C.); 2 Beijing Key Laboratory of Agricultural Genetic Resources and Biotechnology, Institute of Biotechnology, Beijing Academy of Agriculture and Forestry Sciences, Beijing 100097, China; zhanglilina@126.com (L.Z.); mingguiliu@aliyun.com (G.L.)

**Keywords:** transcriptomics, metabolomics, drought stress, rehydration, genes, pathway

## Abstract

Drought stress is a major abiotic factor affecting tomato production and fruit quality. However, the genes and metabolites associated with tomato responses to water deficiency and rehydration are poorly characterized. To identify the functional genes and key metabolic pathways underlying tomato responses to drought stress and recovery, drought-susceptible and drought-tolerant inbred lines underwent transcriptomic and metabolomic analyses. A total of 332 drought-responsive and 491 rehydration-responsive core genes were robustly differentially expressed in both genotypes. The drought-responsive and rehydration-responsive genes were mainly related to photosynthesis–antenna proteins, nitrogen metabolism, plant–pathogen interactions, and the MAPK signaling pathway. Various transcription factors, including homeobox-leucine zipper protein ATHB-12, NAC transcription factor 29, and heat stress transcription factor A-6b-like, may be vital for tomato responses to water status. Moreover, 24,30-dihydroxy-12(13)-enolupinol, caffeoyl hawthorn acid, adenosine 5′-monophosphate, and guanosine were the key metabolites identified in both genotypes under drought and recovery conditions. The combined transcriptomic and metabolomic analysis highlighted the importance of 38 genes involved in metabolic pathways, the biosynthesis of secondary metabolites, the biosynthesis of amino acids, and ABC transporters for tomato responses to water stress. Our results provide valuable clues regarding the molecular basis of drought tolerance and rehydration. The data presented herein may be relevant for genetically improving tomatoes to enhance drought tolerance.

## 1. Introduction

Drought stress, which is one of the most important environmental factors detrimental to sustainable agriculture, is responsible for the largest decreases in global food production [1]. Tomato cultivars are sensitive to drought stress, which can inhibit growth and decrease fruit yield and quality. In contrast, wild tomato resources can grow under extreme drought conditions [2]. Thus, identifying and applying drought tolerance genes from wild tomato germplasm is an effective way to improve the drought tolerance of tomato cultivars. *Solanum pimpinellifolium*, which is the wild tomato species most closely related to *Solanum lycopersicum*, has excellent horticultural traits and is highly drought-tolerant [3,4,5]. Moreover, fertile offspring are produced when it is crossed with *S. lycopersicum*. Therefore, identifying the drought tolerance genes in *S. pimpinellifolium* may be useful for enhancing *S. lycopersicum* drought tolerance.

Drought tolerance is a complex trait regulated by multiple genes and significantly affected by the environment [6,7]. Plants mainly perceive external drought stimuli through membrane sensors, which transmit signals to the corresponding signal transduction pathways, leading to the expression of drought-responsive genes [8,9,10]. Previous studies revealed abscisic acid (ABA) is crucial for drought stress responses because it regulates ABA-responsive signaling components that control stomatal closure and water status, thereby influencing plant adaptations to water deficiency [11,12]. The ABA signaling pathway is the major signaling pathway mediating plant responses to drought stress. Specifically, ABA perceived by the PYR/PYL regulatory components of ABA receptors induces the suppression of PP2Cs and the activation of SnRK2, which phosphorylates downstream targets, including ABA-responsive element-binding factors, bZIP transcription factors, and ion channels, to further regulate ABA-responsive gene expression [13,14,15,16]. The expression of key genes, including signaling-related genes, modulating biosynthesis and catabolism can regulate the endogenous ABA content to mediate plant drought tolerance [17,18]. Additionally, NAC, bZIP, MYB, MYC, and DREB or CBF transcription factors have important regulatory functions related to drought stress responses and recovery [8,19]. Several studies applying RNA-seq technology to clarify the molecular basis of plant drought responses and recovery revealed considerable changes to the expression of genes associated with carbohydrate metabolism, amino acid metabolism, hormone signal transduction, fatty acid metabolism, photosynthesis, and secondary metabolites [20,21,22,23,24].

Metabolomics-based research is critical for studying plant responses to abiotic stresses [25]. The contents of primary and secondary metabolites change during drought stress responses. More specifically, drought conditions sharply increase the abundance of amino acids (e.g., phenylalanine, asparagine, serine, and valine), sugars (e.g., trehalose and glucose), organic acids (e.g., glutaric acid and 2-methylcitric acid), flavonoids (e.g., cyanidin and quercetin), lipids (e.g., acylated steryl glycosides), tricarboxylic acid cycle intermediates (e.g., cis-aconitate and 2-oxoglutarate), ABA, myo-inositol, monoterpenes, and phenylpropanoids, but have the opposite effect on alanine, organic acid (e.g., pyruvate and malonic acid), sugar (e.g., D-galactose and stachyose), nucleoside, and nucleotide (e.g., uridine and guanosine) levels. The metabolites affected by drought vary greatly among plant species [20,24,26,27,28,29,30,31,32,33]. High-throughput techniques for omics-based analyses may be combined to elucidate the mechanisms regulating complex plant traits, including drought stress resistance in tomatoes, which is controlled by diverse factors. Only a few studies have been conducted on tomato drought tolerance and recovery [34,35,36,37,38,39]. Consequently, differences in gene expression and metabolite contents between tomato cultivars and wild species are largely unknown. In this study, transcriptomic and metabolomic analyses of drought-tolerant *S. pimpinellifolium* and drought-sensitive *S. lycopersicum* were performed to identify the functional genes and key metabolic pathways involved in drought stress responses and recovery and to analyze the gene expression and metabolite content changes related to drought stress responses and recovery in *S. pimpinellifolium* and *S. lycopersicum*.

## 2. Results

### 2.1. Phenotypic Characteristics of the Materials Responsive to Drought Stress and Recovery

**Figure 1:**
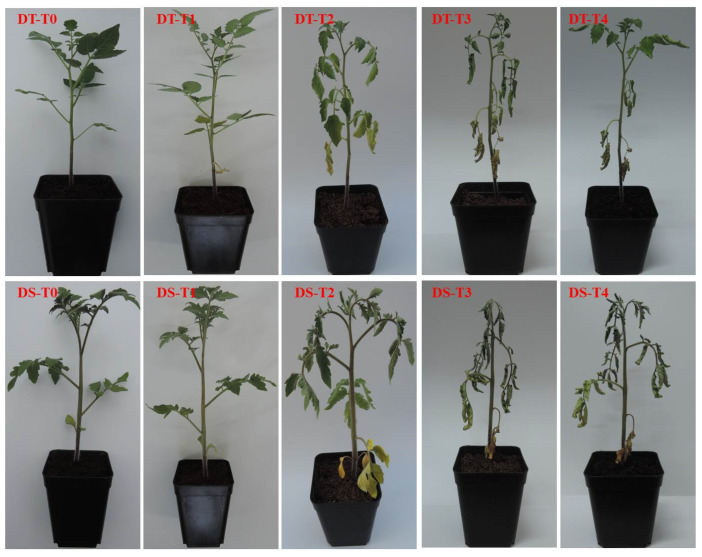
The response of drought-tolerant and susceptible tomatoes to drought stress and dehydration. (DS) represents drought-susceptibility. Moneymaker-1 and DT represent drought-tolerant LA1375-1. T0, T1, T2, and T3 represent the soil moisture reached at 40%, 18%, 12%, and 8% during the drought stress treatment, respectively. T4 represents 1 h after rehydration.

We performed three drought tolerance experiments for LA1375-1 and Moneymaker-1 and found that LA1375-1 was more drought-tolerant than Moneymaker-1 (Table 1). Moneymaker-1 was more sensitive to drought stress than LA1375-1 as the soil moisture gradually decreased, the leaves wilted more quickly, and the leaf wilting degree was heavier. When rehydrated, LA1375-1 recovers faster than Moneymaker-1 (Figure 1).

### 2.2. Transcriptional Characteristics and Core Genes Responsive to Drought Stress and Recovery

Thirty leaf cDNA libraries were sequenced, which yielded more than 6.13 Gb of clean reads per sample, with Q30 scores of 90.50–93.85%. Additionally, 89.73–97.36% of the reads were mapped to the Heinz 1706 genome (Table S1). A total of 27,693 unigenes were obtained, and 19,010 and 18,578 genes were expressed at all time-points (FPKM > 0.1) in drought-susceptible (DS) and drought-tolerant (DT), respectively (Table S2). The FPKM distribution and heatmap revealed many differentially expressed genes (DEGs) among the samples. The expression patterns of the samples with the same genotype were similar at the same time-point (Figures S1 and S2).

**Figure 2:**
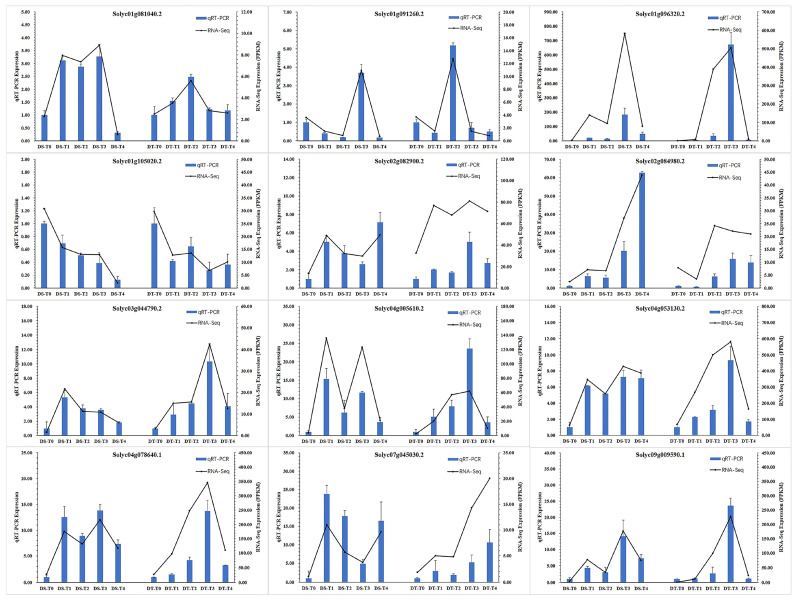
Verification of the DEGs by qRT-PCR. Twelve DEGs were selected for qRT-PCR validation. The relative expression level of each gene was expressed as the FPKM among samples in the RNA-Seq data (black line) and qRT-PCR data (blue bar). The bars represent the standard deviation.

To verify the DEGs identified by RNA-seq, the gene expression patterns of 12 randomly selected DEGs were analyzed by qRT-PCR, using the same samples that were used for RNA-seq analysis. Although the fold-changes of the 12 selected genes differed between the qRT-PCR and RNA-seq data, the expression level trends were the same (Figure 2), reflecting the reliability of the RNA-seq data.

**Figure 3:**
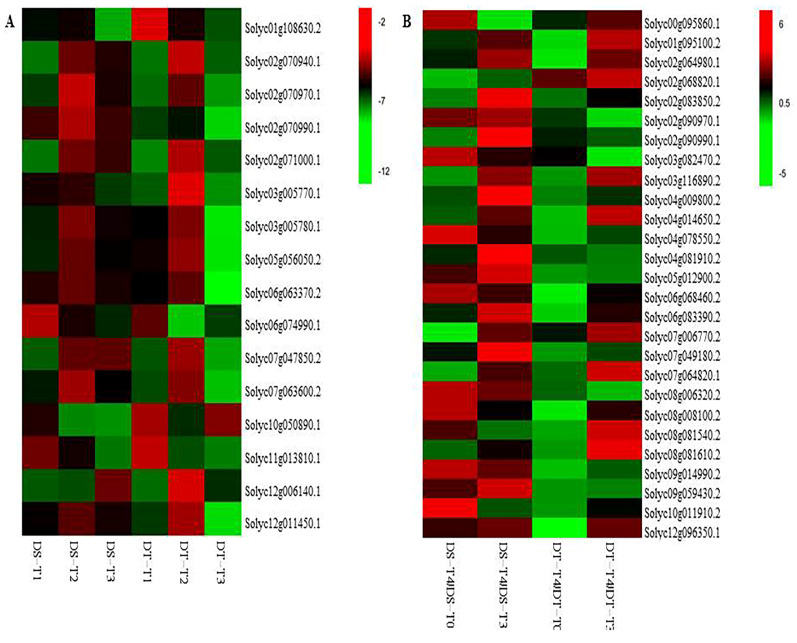
Heatmap of the expression of 16 and 27 genes with enriched GO terms and KEGG pathways in DS and DT under drought and recovery conditions, respectively. (**A**) Heatmap of the expression of 16 genes with enriched GO terms and KEGG pathways in DS and DT under drought conditions. The bar represents the log_2_(fold-change) of each gene relative to the control level in DS-T1, DS-T2, DS-T3, DT-T1, DT-T2, and DT-T3. (**B**) Heatmap of the expression of 27 genes with enriched GO terms and KEGG pathways in DS and DT under recovery conditions. The bar represents the log_2_(fold-change) of each gene in DS-T4/DS-T0, DS-T4/DS-T3, DT-T4/DT-T0, and DT-T4/DT-T3.

A comparison of the gene expression at the three sample collection time-points during the drought treatment revealed 897 and 1876 DEGs in DS and DT, respectively (Figure S3A,B). Specifically, 502 and 1416 genes were downregulated, whereas 381 and 403 genes were upregulated in DS and DT, respectively. To identify the core genes involved in tomato responses to drought stress, the overlapping genes from among 883 and 1819 genes with consistently regulated changes to expression in DS and DT, respectively, were analyzed further. The most significantly enriched GO terms among the 332 overlapping DEGs between the two genotypes (Figure S3C, Table S3) were “photosynthesis, light harvesting” and “photosynthesis, light harvesting in photosystem I” (corrected *p* = 3.54 × 10^−13^) in the biological process group, “photosystem I” (corrected *p* = 4.07 × 10^−12^) in the cellular component group, and “pigment binding” (corrected *p* = 2.13 × 10^−11^) in the molecular function group (Table S4). The significantly enriched GO terms were assigned to 51 downregulated genes (e.g., chlorophyll a-b binding protein, aspartyl protease family protein 2, and trehalose-phosphate phosphatase A) and 13 upregulated genes (e.g., stress enhanced protein 2, trehalose-6-phosphate synthase, and transcription factor JUNGBRUNNEN 1-like isoform X1). The significantly enriched KEGG pathways for 12 and 5 genes were “photosynthesis–antenna proteins” (corrected *p* = 7.52 × 10^−12^) and “nitrogen metabolism” (corrected *p* = 0.022), respectively (Table S5). The expression of these genes was downregulated compared with the control level. Moreover, with the exception of *Solyc03g083440.2* (glutamate synthase 1), these genes were annotated with GO terms (Figure 3A, Tables S7 and S8).

The four comparisons of gene expression levels to detect the DEGs induced by the recovery treatment indicated that 2546 and 2306 different genes in DS-T4 were consistently downregulated and upregulated relative to the DS-T0 and DS-T3 levels, respectively, whereas 770 and 101 different genes in DT-T4 were consistently downregulated and upregulated relative to the DT-T0 and DT-T3 levels, respectively. A total of 491 genes overlapped between the two genotypes (Figure S3D, Table S7). The most significantly enriched GO terms were “response to chitin” (corrected *p* = 1.58 × 10^−12^) in the biological process group and “signaling receptor binding” (corrected *p* = 5.13 × 10^−4^) in the molecular function group (Table S7). Significantly enriched GO terms were assigned to 152 DEGs identified under recovery conditions. Of these genes, 91 were annotated with multiple GO terms, and 61 were annotated with only one GO term (Table S7). The significantly enriched KEGG pathways for 32 and 24 genes were “plant–pathogen interaction” (corrected *p* = 4.50 × 10^−6^) and “MAPK signaling pathway–plant” (corrected *p* = 1.26 × 10^−4^), respectively, and 14 genes overlapped (Table S8). Furthermore, enriched GO terms and KEGG pathways were identified for 27 genes, all of which were upregulated, except for *Solyc02g068820.1*, and were annotated as LRR receptor-like serine/threonine-protein kinase EFR genes that were downregulated in DS and upregulated in DT (Figure 3B).

### 2.3. Transcription Factors Are Involved in Drought Stress Responses and Recovery

**Figure 4:**
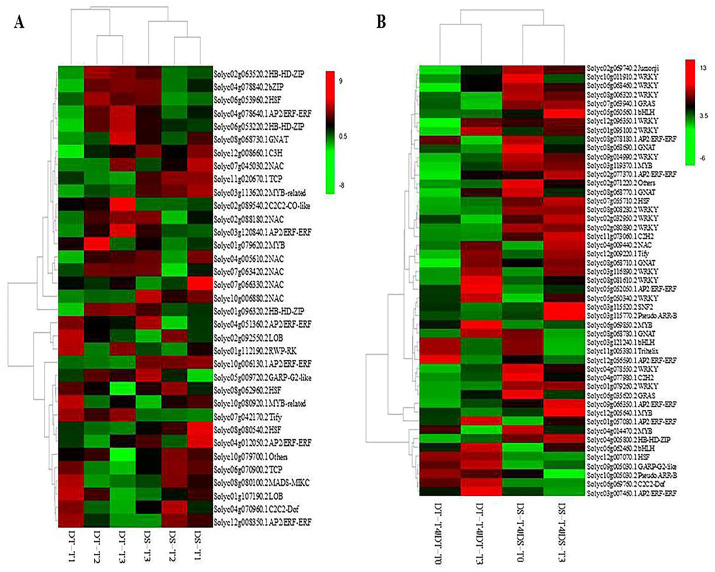
Heatmap of the expression of 35 and 49 differentially co-expressed transcription factor genes in DS and DT under drought conditions and responsive to rehydration, respectively. (**A**) Heatmap of the expression of 35 differentially co-expressed transcription factor genes in DS and DT under drought conditions. The bar represents the fold-change of each gene, relative to the control level, in DS-T1, DS-T2, DS-T3, DT-T1, DT-T2, and DT-T3. Red and green indicate upregulated and downregulated transcription factor gene expression, respectively. (**B**) Heatmap of the expression of 49 differentially co-expressed transcription factor genes in DS and DT responsive to rehydration. The bar represents the fold-change of each gene relative to the control and T3 levels in DS-T4 and DT-T4. Red and green indicate upregulated and downregulated transcription factor gene expression, respectively.

There were 117 and 162 common differentially expressed transcription factors in DS and DT, respectively, under drought conditions, and 35 transcription factor genes overlapped (Figure 4A). Transcription factor genes in the NAC, HB-HD-ZIP, bZIP, GNAT, C3H, MYB, and C2C2-CO-like families were usually upregulated, unlike those in the LOB, RWP-RK, GARP-G2-like, HSF, Tify, MADS-MIKC, and C2C2-Dof families, which were usually downregulated (Figure 4A). The “circadian rhythm–plant” (corrected *p* = 9.39 × 10^−4^) and “plant hormone signal transduction” (corrected *p* = 9.54 × 10^−3^) were enriched KEGG pathways for three and four genes, respectively (Table S9).

After rehydration, there were 228 and 216 common differentially expressed transcription factors in DS and DT, respectively, and 68 transcription factor genes in common and 49 exhibited the same expression trend after rehydration in the two genotypes (41 upregulated and 8 downregulated). The upregulated transcription factor genes mainly included WRKY, AP2/ERF-ERF, GNAT, and MYB family members (Figure 4B). Conversely, the downregulated transcription factor genes mainly included AP2/ERF-ERF, bHLH, and C2C2-Dof family members (Figure 4B). The enriched KEGG pathways for 12, 8, 3, and 6 genes were respectively “plant–pathogen interaction” (corrected *p* = 1.60 × 10^−7^), “MAPK signaling pathway–plant” (corrected *p* = 1.28 × 10^−4^), “circadian rhythm–plant” (corrected *p* = 0.032), and “plant hormone signal transduction” (corrected *p* = 0.037) (Table S10).

The transcription factor genes encoding homeobox-leucine zipper protein ATHB-12 (*Solyc01g096320.2*), NAC transcription factor 29 (*Solyc04g005610.2*), and heat stress transcription factor A-6b-like (*Solyc06g053960.2*) were involved in both drought stress responses and recovery. The expression of these genes was upregulated in DS under drought and recovery conditions but was upregulated under drought conditions and downregulated under recovery conditions in DT, suggesting these genes play key roles in tomato drought stress responses and recovery.

### 2.4. Core Metabolites Affected by Drought Stress and Recovery

A total of 553 unique metabolites were identified by a global untargeted metabolite analysis using the UPLC and MS/MS platforms. The water status-responsive compounds were mainly flavonoids (125), phenolic acids (85), and amino acids and derivatives (72) (Table S11). The DS and DT samples were clustered together, and the three biological replicates of each sample were clustered in a group, except for DS-T1 and DS-T2 (Figure S4). 

To determine the core metabolites involved in drought stress responses, we compared the metabolites in T1, T2, and T3 between DS and DT to identify the common differentially abundant metabolites. Compared with DS-T0, the abundance of 113 and 9 metabolites was consistently higher and lower, respectively, in DS-T1, DS-T2, and DS-T3 (Figure S5A, Table S12). Compared with DT-T0, the contents of one and four metabolites were consistently higher and lower, respectively, in DT-T1, DT-T2, and DT-T3 (Figure S5B, Table S13). Under drought conditions, 24,30-dihydroxy-12(13)-enolupinol and caffeoyl hawthorn acid were the common differentially abundant metabolites in DS and DT, and the contents of both substantially increased relative to the control levels, although the changes following different treatments were much greater in DT than in DS (Tables S17 and S18). This suggests that they likely positively regulate drought stress responses and are the key metabolites associated with the differences between DS and DT in response to water status. 

After rehydration, the contents of 8 and 30 different metabolites were consistently lower and higher, respectively, in DS-T4 than in DS-T0 and DS-T3. The overlapping metabolites between DS and DT were adenosine 5′-monophosphate and guanosine (Tables S14 and S15). The abundance of both decreased, implying they are most likely the key metabolites associated with drought stress recovery.

### 2.5. Important Metabolic Pathways Involved in Drought Responses and Recovery

**Figure 5:**
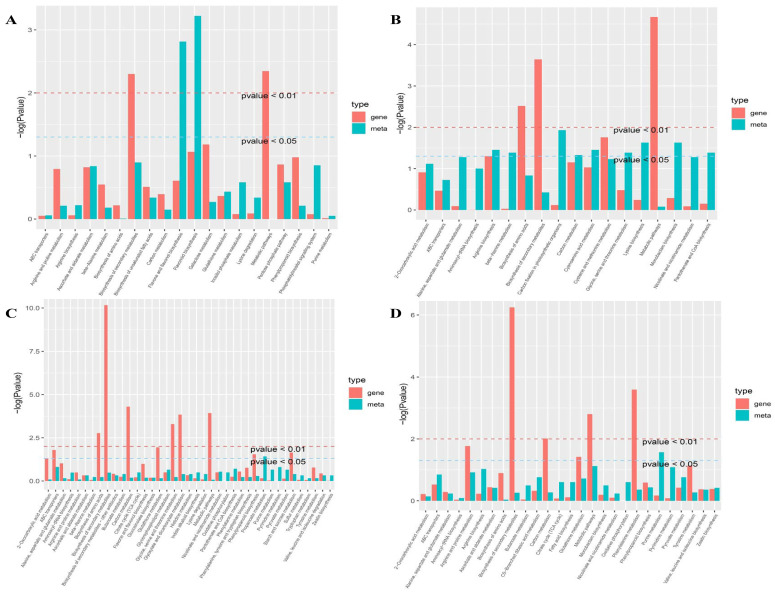
*p* value histograms for the KEGG enrichment analysis under drought conditions and during rehydration. (**A**) *p* value histogram for the KEGG enrichment analysis of DS under drought conditions. (**B**) *p* value histogram for the KEGG enrichment analysis of DT under drought conditions. (**C**) *p* value histogram for the KEGG enrichment analysis of DS during rehydration. (**D**) *p* value histogram for the KEGG enrichment analysis of DT during rehydration. The abscissa of the histograms presents the metabolic pathways. Red and green represent the enriched *p* values for the differentially expressed genes and differentially abundant metabolites, respectively.

In a joint transcriptome and metabolome analysis, 109 DEGs and 87 metabolites were mapped to different steps of 380 pathways, and 31 DEGs and 4 metabolites were mapped to different steps of 39 pathways (correlation coefficient > 0.8) among the drought-responsive core DEGs and metabolites in DS and DT, respectively (Figure 5A,B). Additionally, 20 and 18 KEGG pathways were enriched in DS and DT, respectively, under drought conditions, of which metabolic pathways, biosynthesis of secondary metabolites, carbon metabolism, biosynthesis of amino acids, arginine biosynthesis, beta-alanine metabolism, and ABC transporters were enriched in both genotypes. Interestingly, only one metabolite, L-aspartic acid, was involved in all 18 pathways in DT (Tables S16 and S17).

After rehydration, 1743 DEGs and 37 metabolites in DS were mapped to different steps of 3285 pathways, whereas 524 DEGs and 10 metabolites in DT were mapped to different steps of 616 pathways (correlation coefficient > 0.8). Additionally, 39 and 27 KEGG pathways were enriched in DS and DT, respectively (Figure 5C,D), with 22 pathways enriched in both genotypes, including phenylpropanoid biosynthesis, ascorbate and aldarate metabolism, pyruvate metabolism, glutathione metabolism, ABC transporters, and amino acid metabolism-related pathways (e.g., biosynthesis of amino acids; alanine, aspartate, and glutamate metabolism; arginine and proline metabolism; phenylalanine metabolism; and tyrosine metabolism) (Tables S18 and S19).

**Figure 6:**
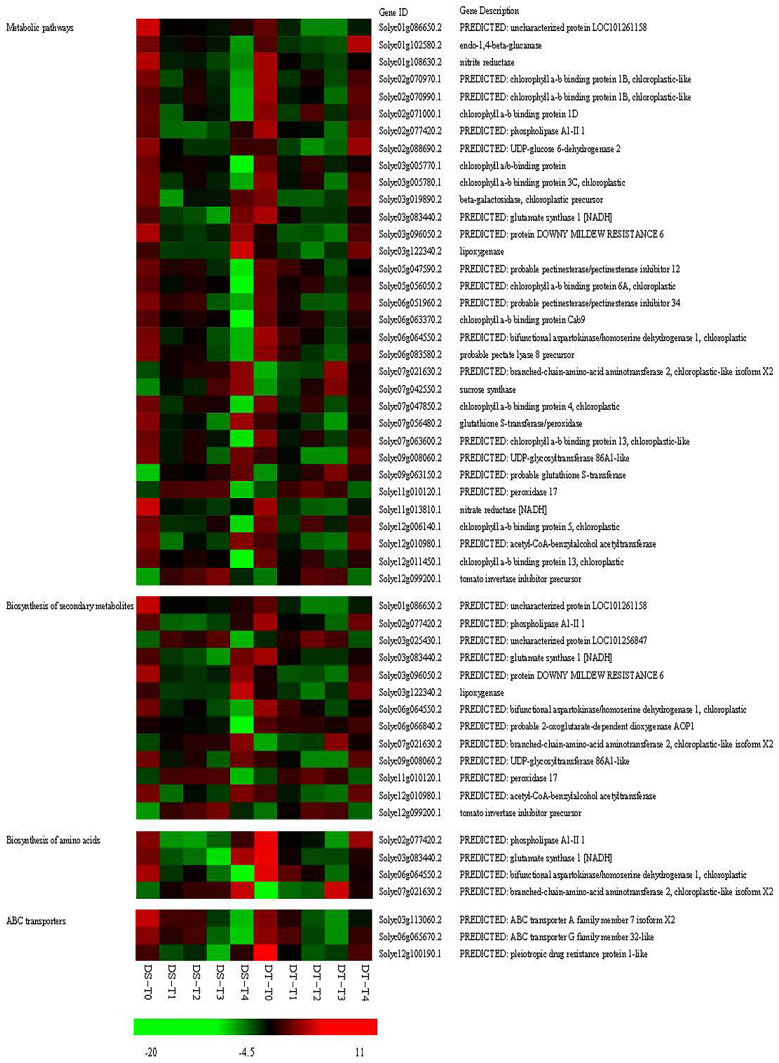
Heatmap of the expression of overlapping genes with enriched KEGG pathways in DS and DT under drought and recovery conditions. The bar represents the log_2_ FPKM of each gene. Red and green indicate upregulated and downregulated gene expression, respectively.

Further analyses indicated that five KEGG pathways (biosynthesis of amino acids, biosynthesis of secondary metabolites, carbon metabolism, metabolic pathways, and ABC transporters) were enriched in both genotypes under drought and recovery conditions (Figure 5A–D). Moreover, 38 genes overlapped between DS and DT under drought and recovery conditions, of which 33 genes were related to metabolic pathways, 13 genes were related to the biosynthesis of secondary metabolites, 4 genes were related to the biosynthesis of amino acids, and 3 genes were related to ABC transporters (Figure 6), suggesting these genes are important hub genes for tomato responses to water status. Furthermore, four genes (*Solyc02g077420.2*, *Solyc03g083440.2*, *Solyc06g064550.2*, and *Solyc07g021630.2*) associated with the biosynthesis of amino acids were also related to metabolic pathways and the biosynthesis of secondary metabolites (Figure 6), implying amino acids are important for drought stress responses and rehydration.

### 2.6. Identification of Key Genes and Pathways

**Figure 7:**
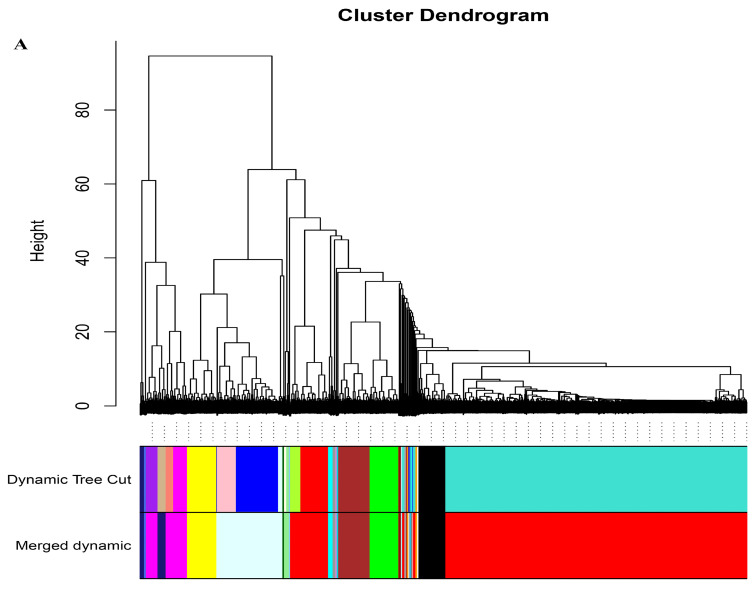

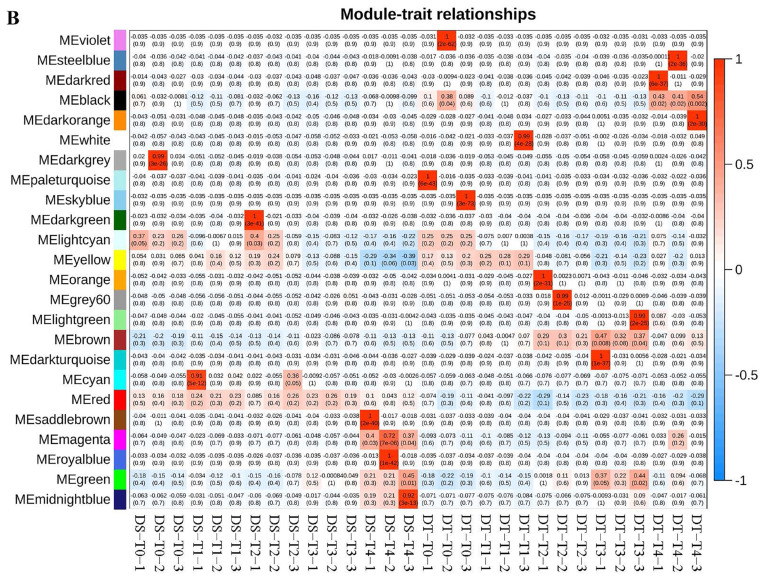

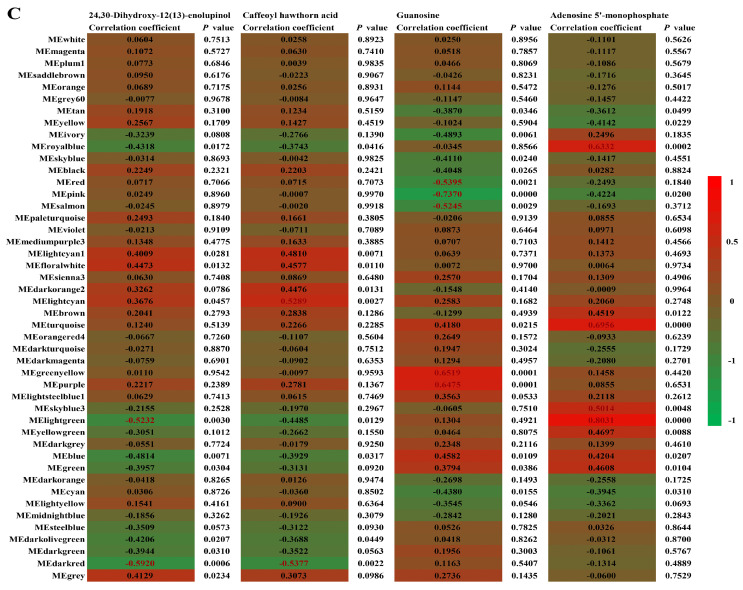
WGCNA of genes and drought stress and rehydration traits. (**A**) Cluster tree based on the WGCNA. The tree branches into 24 modules with various colors. (**B**) Module–trait correlations. Each row corresponds to a module, which is colored as in (**A**). The color at the row–column intersection reflects the correlation between the module and trait. (**C**) Module correlations for 24,30-dihydroxy-12(13)-enolupinol, caffeoyl hawthorn acid, adenosine 5′-monophosphate, and guanosine. Each row corresponds to a module eigengene (the correlation between a column and a trait). Each cell contains the corresponding correlation and *p* value. The strength of a correlation is indicated by color.

We performed a weighted gene correlation network analysis (WGCNA) of all unigenes to comprehensively characterize the expression of genes responsive to drought stress and rehydration. Twenty-four modules were identified (Figure 7A). The brown module was positively correlated with drought stress in DT, whereas the magenta and black modules were positively correlated with rehydration in DS and DT, respectively (*p* < 0.05) (Figure 7B). The top 10 hub genes in each module and their kME (eigengene connectivity) values are listed in Table 2. On the basis of the DEG analysis, genes were annotated as serine/threonine protein kinase, peroxidase 63, ABC transporter, and transcription factor MBF1. The enriched KEGG pathways were MAPK signaling pathway–plant and plant–pathogen interaction for the black and magenta modules, but autophagy–other and spliceosome for the brown module. Therefore, genes related to the MAPK signaling pathway are important for tomato responses to water stress. Additionally, several other KEGG pathways were also enriched, including plant hormone signal transduction, cutin, suberine, and wax biosynthesis, and fatty acid elongation in the black module; SNARE interactions in vesicular transport, N-glycan biosynthesis, ubiquitin-mediated proteolysis, pantothenate and CoA biosynthesis, and arachidonic acid metabolism in the brown module; and alpha-linolenic acid and glycerophospholipid metabolism in the magenta module (Table S20).

Forty-six modules were identified based on the WGCNA of the unigenes and the common differentially abundant metabolites 24,30-dihydroxy-12(13)-enolupinol, caffeoyl hawthorn acid, adenosine 5′-monophosphate, and guanosine. More specifically, 24,30-dihydroxy-12(13)-enolupinol was significantly correlated with the dark red and light green (R < −0.50 and *p* < 0.001) modules (Figure 7C). Caffeoyl hawthorn acid was highly associated with the dark red and light cyan modules (R < −0.50 or > 0.5 and *p* < 0.001), adenosine 5′-monophosphate was highly associated with the light green module (R > 0.8 and *p* < 0.001), and guanosine was highly associated with the pink module (R < −0.7 and *p* < 0.001). The KEGG analysis indicated the light green module was mainly related to plant hormone signal transduction, the dark red module was mainly related to biosynthesis of secondary metabolites, sesquiterpenoid and triterpenoid biosynthesis, selenocompound metabolism, and photosynthesis–antenna proteins, the light cyan module was mainly related to phenylpropanoid biosynthesis and MAPK signaling pathway–plant, the turquoise module was mainly related to RNA transport, mRNA surveillance pathway, RNA degradation, and lysine degradation, the royal blue module was mainly related to zeatin biosynthesis, the pink module was mainly related to plant–pathogen interaction, endocytosis, and MAPK signaling pathway–plant, and the purple module was mainly related to carbon metabolism, carbon fixation in photosynthetic organisms, and pyruvate metabolism (corrected *p* < 0.05) (Table S21). Thus, the genes related to these pathways are likely correlated with water stress responses, and their expression is regulated at the transcriptional level under drought and recovery conditions.

## 3. Discussion

We conducted transcriptomic and metabolic analyses of leaves collected from *S. pimpinellifolium* and *S. lycopersicum* plants exposed to gradually increasing drought stress and then rehydrated. In response to increasing drought stress, more genes with upregulated or downregulated expression levels were detected in DT than in DS. In contrast, more genes were responsive to rehydration in DS than in DT. The metabolite contents changed more in DS than in DT in response to drought conditions and rehydration. These findings suggest the drought-resistant genotype was more affected by drought stress at the transcriptional level, whereas the drought-susceptible genotype was more affected by rehydration at the transcriptional level and by drought stress and rehydration at the metabolic level. We speculate that the drought-susceptible genotype lacks a homeostatic mechanism that can alleviate the effects of water deficiency and rehydration. Our results differ slightly from those of earlier studies on drought-sensitive crops (e.g., rice, barley, banana, and sesame) that are more susceptible to water stress at molecular and metabolic levels [20,24,40,41].

### 3.1. Reactive Oxygen Species-Related Genes and Metabolites Are Involved in Drought Stress Responses and Recovery

In plants, the cellular ROS content is normally maintained by balancing ROS production and elimination via a regulatory network involving many genes, proteins, and other molecules. The elimination of ROS is mainly achieved through complex non-enzymatic and enzymatic antioxidant systems in plants. Non-enzymatic antioxidants mainly include vitamin E, vitamin C, reduced glutathione, β-carotene, and osmolytes (e.g., proline, betaine, and soluble sugars). The primary enzymatic antioxidants are superoxide dismutase, ascorbate peroxidase, glutathione peroxidase, and catalase [42]. Under abiotic stress conditions, ROS significantly accumulate, resulting in oxidative damage to plant cells [24,43,44]. In this study, the expression of genes encoding ROS-scavenging enzymes, such as superoxide dismutase, glutathione peroxidase, ascorbate peroxidase, peroxiredoxin, and catalase, was significantly modulated by drought stress and rehydration, with important implications for ROS homeostasis and drought resistance [24,44,45,46]. Glutathione S-transferase (GST) is crucial for glutathione metabolism, which makes an important contribution to plant abiotic stress resistance and cellular redox homeostasis [47,48,49]. In this study, several drought-inducible GST genes were identified, with higher expression levels in DT than in DS under drought conditions. The expression levels of some GST genes were affected by rehydration, suggesting that GST-encoding genes are closely related to drought resistance and rehydration in tomatoes. Some of the upregulated genes were mapped to antioxidant metabolic pathways under drought conditions, including phenylpropanoid biosynthesis, carotenoid biosynthesis, peroxisome, and pyruvate metabolism. Moreover, proline and soluble sugars are osmolytes and free-radical scavengers that accumulate in response to stress [50,51,52]. In the current study, proline and soluble sugar contents changed significantly in the two genotypes under drought and rehydration conditions (Table S11). Therefore, ROS-related genes and metabolites play an important role in tomato responses to water status.

### 3.2. Amino Acid Metabolic Pathways Are Highly Responsive to Drought Stress and Rehydration

Specific amino acids are essential for plant stress tolerance because they can function as ROS scavengers, osmolytes, signaling molecules, and the precursors of energy-associated metabolites [24,50,53,54]. In this study, large amounts of amino acids accumulated in both genotypes under drought conditions, which was consistent with the results of previous research on different plant species [24,26,29,31]. Our transcriptomic and metabolic profile analyses revealed that many genes and metabolites associated with amino acid metabolism were responsive to drought stress and rehydration. The amino acids mainly included serine, citrulline, aspartic acid, tryptophan, histidine, proline, arginine, isoleucine, lysine, cysteine, alanine, glutamate, glycine, threonine, and cyanoamino acid. Aldehyde dehydrogenase (NAD^+^) (K00128) is the key enzyme, and 5-aminovaleric acid and spermidine are important metabolites in the arginine and proline metabolic pathways. In this study, the expression of aldehyde dehydrogenase-encoding genes (*Solyc06g064900.2*, *Solyc11g071550.1*, *Solyc03g098300.1*, *Solyc01g087590.2*, and *Solyc02g081390.2*) and the contents of 5-aminovaleric acid and spermidine changed significantly during the drought and rehydration treatments. Moreover, *Solyc06g064900.2* and *Solyc12g008680.1*, which are aldehyde dehydrogenase genes, and 5-aminovaleric acid involved in the lysine degradation pathway were greatly affected by drought stress and rehydration. Therefore, significant changes to amino acid metabolic pathways are among the important characteristics of tomato responses to drought and rehydration.

### 3.3. Genes Involved in ABA Metabolism and Signaling Are Crucial for Responses to Water Status

**Figure 8:**
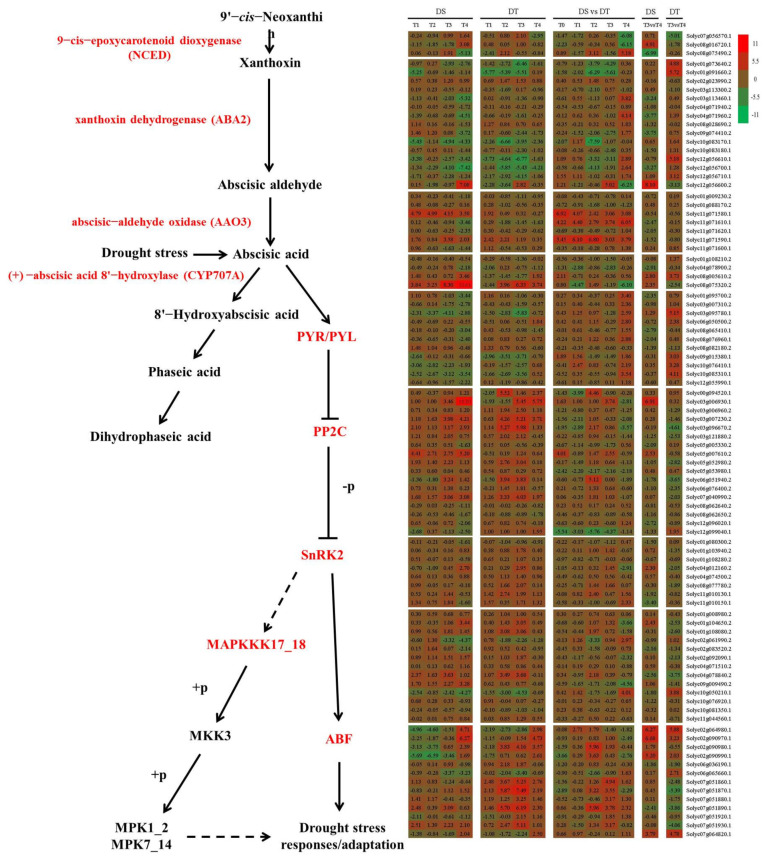
Schematic of ABA metabolism in DS and DT under drought and recovery conditions. The bar represents the log_2_ (fold change) of each gene. Red and green indicate upregulated and downregulated gene expression, respectively.

Phytohormones regulate plant protective responses to various environmental stresses through complex regulatory networks that integrate external stimuli. Abscisic acid is a key phytohormone in plant adaptive responses to drought stress. Thus, moisture status-induced changes to ABA contents in plant cells may regulate the activation of ABA-responsive genes and the stomatal state to minimize water loss [12,16,55]. In the current study, the expression patterns of genes involved in ABA metabolism and signaling during drought stress and rehydration changed markedly in the two genotypes (Figure 8). The biosynthesis of ABA requires 9-cis-epoxycarotenoid dioxygenase (NCED), xanthoxin dehydrogenase (ABA2), and abscisic-aldehyde oxidase (AAO3). Our gene expression data indicated that the expression of 3 genes encoding NCED, 15 genes encoding ABA2, and 7 genes encoding AAO3 changed in response to drought stress and rehydration in the two genotypes. The hydroxylation of ABA, which is a critical step during ABA catabolism, is catalyzed by the (+)-abscisic acid 8′-hydroxylase (CYP707A). Four genes annotated as abscisic acid 8′-hydroxylases responded differently to drought stress and rehydration. Moreover, PYR/PYL, PP2C, SnRK2, and ABF are believed to be important for ABA signal transduction related to plant responses to drought stress. Furthermore, PYR/PYL, PP2C, SnRK2, MAPKKK17_18, MKK3, and MPK1_2 are crucial for drought stress responses involving the MAPK signaling pathway. In this study, the expression of 11 genes encoding PYR/PYL, 17 genes encoding PP2C, 8 genes encoding SnRK2, 13 genes encoding ABF, 13 genes encoding MAPKKK17_18, 1 gene encoding MKK3, and 1 gene encoding MPK1_2 changed considerably during the drought treatment period and rehydration. These results suggest ABA metabolism and signaling are vital for tomato responses to water status, which is consistent with the findings of earlier investigations of other plants [11,14,16,24,56,57,58].

### 3.4. Transcription Factors Associated with Responses to Water Stress

Transcription factors are important regulators of gene expression during plant development and abiotic stress responses. In this study, the transcription factors homeobox-leucine zipper protein ATHB-12, NAC transcription factor 29, and heat stress transcription factor A-6b-like were simultaneously involved in drought stress responses and rehydration. The corresponding genes were differentially expressed between the treated and control samples in both genotypes. Interestingly, these genes were similarly expressed. Their expression levels were upregulated by drought stress in both genotypes, but they were upregulated in DS and downregulated in DT after the rehydration. Homeobox-leucine zipper protein ATHB-12 is a probable transcriptional activator that may regulate growth in response to drought and ABA [59,60,61,62]. A previous study identified NAC transcription factor 29 as a transcriptional activator that binds to and activates the *AAO3* promoter, thereby inducing chlorophyll degradation in leaves and leading to increased levels of the senescence-inducing hormone ABA [58]. It is also involved in controlling the dehydration of senescent leaves, binds to the *SAG113* promoter to negatively regulate ABA signaling for mediating stomatal closure in leaves, controls water loss during leaf senescence, and delays leaf senescence. There were no obvious phenotypic differences under normal growth conditions, but mutant leaves exhibit a stay-green phenotype and a deficiency in chlorophyll degradation during extended periods of darkness. Moreover, the expression levels of NAC family transcription factor genes involved in senescence, nitrogen-associated metabolism, and growth are upregulated in senescing leaves because of the inductive effects of ABA and ethylene [58,63,64,65]. Heat stress transcription factor A-6b-like positively regulates the transcription of RNA polymerase II in response to abiotic stresses, including heat [66]. Therefore, transcription factors, especially ABA-responsive signaling components, have key roles in the complex networks regulating tomato responses to water status. The expression of transcription factor-encoding genes (e.g., NAC, HB-HD-ZIP, AP2/ERF-ERF, bZIP, GNAT, MYB, HSF, MADS-MIKC, WRKY, bHLH, GARP-G2-like, and C2H2 family members) changed substantially under drought and recovery conditions in this study, which is in accordance with the observations of earlier investigations [8,19,67].

## 4. Materials and Methods

### 4.1. Plant Materials

*Solanum lycopersicum* cv. Moneymaker and *S. pimpinellifolium* LA1375 seeds were obtained from the Tomato Genetics Resource Center at the University of California (Davis, CA, USA). We then obtained drought-susceptible (DS) Moneymaker-1 and drought-tolerant (DT) LA1375-1 lines through screening and purification (*n* > 6) [68]. Plants were grown in pots (7 cm diameter and 7.5 cm depth) containing 40 g of nutrient soil in a greenhouse in Haidian (Beijing, China). Plants were exposed to drought stress at the five-leaf stage. Soil moisture was monitored throughout the study period using the VM-220 High Frequency Moisture Meter (Spike Instrument Technology Co., Ltd., Hefei, An’hui, China). Drought stress treatment and investigation standards were performed according to Liu’s method [68]. The fourth leaf was collected from three randomly selected plants (as one biological replicate) when the soil moisture reached 40% (T0, saturated water content), 18% (T1), 12% (T2), and 8% (T3) during the drought stress treatment, as well as at 1 h after rehydration (T4). Leaf samples were collected between 15:30 and 16:00. Three replicates of Moneymaker-1 and LA1375-1 leaf samples (T0–T4) were used for RNA extraction and metabolite analysis.

### 4.2. RNA Extraction and Illumina Sequencing

Total RNA was extracted from leaves using the EASYspin Plus Plant RNA Kit (Jingchangkeyi Co., Ltd., Beijing, China). The RNA purity and integrity were evaluated by agarose gel electrophoresis. Additionally, the RNA concentration and integrity were determined using the Qubit^®^ RNA Assay Kit and the Qubit^®^ 2.0 Fluorometer (Life Technologies Corporation, Carlsbad, CA, USA), as well as the RNA Nano 6000 Assay Kit and the Bioanalyzer 2100 system (Agilent Technologies Inc., Santa Clara, CA, USA). The RNA-seq libraries were constructed from cDNA fragments (300–350 bp long) and clustered using the Illumina TruSeq PE Cluster Kit (v3-cBot-HS) and the cBot Cluster Generation System. The libraries were sequenced on the Illumina HiSeq™ 4000 system (150 bp paired-end reads).

### 4.3. Genome Mapping and Differential Gene Expression Analysis

We obtained clean reads by removing the adapters and low-quality reads (i.e., reads with more than 10% unknown nucleotides or more than 50% bases with a Q-value ≤ 20) from the raw data. The Q20, Q30, and GC content were calculated based on the clean reads, which were then mapped to the tomato Heinz 1706 reference genome (https://solgenomics.net/organism/Solanum_lycopersicum/genome, accessed on 25 November 2019) using HISAT2.1.0 [69].

The fragments per kilobase of transcript per million fragments mapped (FPKM) value [70] was used to calculate and normalize gene expression levels. A quantitative real-time polymerase chain reaction (qRT-PCR) assay was used to validate the expression levels of 12 randomly selected genes as previously described [71]. The tomato actin gene *Solyc03g078400* was used as an internal control to normalize gene expression data. Details regarding the PCR primers are provided in Table S22. Differential gene expression was analyzed using DESeq2 [72]. The *p* values were adjusted to control the false discovery rate (FDR) according to Benjamini and Hochberg’s method [73]. Significantly differentially expressed genes (DEGs) were determined based on the following threshold criteria: FDR < 0.05 and |log_2_(fold-change)| ≥ 1. The GOseq2.12 [74] and KOBAS 2.0 software [75] were respectively used to complete the Gene Ontology (GO) and Kyoto Encyclopedia of Genes and Genomes (KEGG) pathway enrichment analyses of the DEGs according to the Wallenius non-central hypergeometric distribution [76]. Transcription factors were identified based on a database search using hmmscan and the iTAK1.5 software.

### 4.4. Untargeted Metabolomics Analysis

An UPLC (Shim-pack UFLC SHIMADZU CBM30A, Shanghai, China) and MS/MS (Applied Biosystems 4500 QTRAP, Foster City, CA, USA) platform was used for the untargeted metabolomics profiling, operation, and data processing methods as previously described [77]. To compare the metabolite contents of different samples, we calibrated the mass spectrum peaks for each metabolite in the analyzed samples based on the retention times and peak types. Multivariate statistical methods of OPLS-DA were used to maximize the metabolomic differences among samples. The relative importance of each metabolite to the OPLS-DA model was evaluated using the VIP scores. Metabolites with a fold-change ≥ 2.0 or ≤0.50 and a VIP score ≥ 1.0 were considered to be significantly differentially abundant metabolites. The identified metabolites were annotated based on the KEGG compound database and assigned to KEGG pathways (http://www.kegg.jp/kegg/, accessed on 29 December, 2019). The significance of the enrichment of these pathways was determined based on hypergeometric test *p* values.

### 4.5. Weighted Gene Correlation Network Analysis (WGCNA)

Weighted gene correlation network analysis was constructed using the WGCNA package in the R software 4.2.1 with the default parameters. All unigenes were further divided into twenty-four modules using WGCNA, and the correlation of each module with drought and stress was calculated. Module-trait associations were estimated using the correlation between the module eigengene and drought stress and rehydration treatments. 

## 5. Conclusions

We comprehensively and systematically analyzed the global transcriptional and metabolic changes in *S. pimpinellifolium* and *S. lycopersicum* under drought and recovery conditions. The genes responsive to drought and rehydration were mainly associated with photosynthesis–antenna proteins and nitrogen metabolism, as well as plant–pathogen interactions and the MAPK signaling pathway. Transcription factors, including homeobox-leucine zipper protein ATHB-12, NAC transcription factor 29, and heat stress transcription factor A-6b-like, may have vital functions during responses to water status. Additionally, 24,30-dihydroxy-12 (13)-enolupinol, caffeoyl hawthorn acid, adenosine 5′-monophosphate, and guanosine are the key metabolites in both genotypes under drought and recovery conditions. By combining transcriptomic and metabolomic analyses, we identified 38 genes involved in metabolic pathways, the biosynthesis of secondary metabolites, the biosynthesis of amino acids, and ABC transporters related to responses to water stress. Furthermore, genes and metabolites related to ROS, amino acid metabolism, and ABA metabolism and signaling are crucial for tomato drought stress responses and rehydration. The water status-responsive genes and metabolic pathways identified in this study will help clarify plant drought tolerance and rehydration at the molecular and metabolic levels. The presented findings will also be useful for breeding drought-tolerant tomato cultivars.

## Figures and Tables

**Table 1:** Drought tolerance scales of LA1375-1 and Moneymaker-1.

Code	Experiment 1	Experiment 2	Experiment 3
LA1375-1	2.58 ± 0.12 b	1.58 ± 0.14 b	1.92 ± 0.14 b
Moneymaker-1	4.88 ± 0.18 a	4.33 ± 0.14 a	4.78 ± 0.19 a

Values followed by the same letters are not significantly different at *p* = 0.01, based on Duncan’s Multiple Range Test.

**Table 2:** Hub genes of WGCNA analysis.

Gene ID	Module	kME	*p* Value	Annotation
*Solyc12g006970.1*	black	0.9677	2.76 × 10^−18^	--
*Solyc02g087960.2*	black	0.9600	5.18 × 10^−17^	Myb-related protein 306-like; Transcription factor, Myb superfamily
*Solyc03g097050.2*	black	0.9553	2.39 × 10^−16^	Cellulose synthase-like protein D3
*Solyc07g066100.2*	black	0.9527	5.10 × 10^−16^	GPI-anchored protein LORELEI
*Solyc03g026270.1*	black	0.9512	7.96 × 10^−16^	Dehydration-responsive element-binding protein 1A-like, CRT binding factor 3
*Solyc02g087190.1*	black	0.9501	1.08 × 10^−15^	Peroxidase 63
*Solyc01g057770.2*	black	0.9497	1.19 × 10^−15^	Boron transporter 1 isoform X2, Na+-independent Cl/HCO3 exchanger AE1 and related transporters (SLC4 family)
*Solyc04g079120.2*	black	0.9474	2.21 × 10^−15^	Probable protein phosphatase 2C 12, Serine/threonine protein phosphatase
*Solyc10g074540.1*	black	0.9448	4.22 × 10^−15^	Protein EXORDIUM-like 5
*Solyc04g074410.1*	black	0.9422	7.95 × 10^−15^	Protein EXORDIUM-like
*Solyc07g061960.2*	brown	0.9782	1.17 × 10^−20^	Persulfide dioxygenase ETHE1 homolog, mitochondrial isoform X1, Glyoxylase
*Solyc06g007330.2*	brown	0.9639	1.25 × 10^−17^	GILT-like protein F37H8.5, Gamma-interferon inducible lysosomal thiol reductase
*Solyc09g089650.1*	brown	0.9610	3.66 × 10^−17^	Uncharacterized protein At4g14450, chloroplastic-like
*Solyc10g007350.2*	brown	0.9558	2.03 × 10^−16^	Multiprotein bridging factor 1, Transcription factor MBF1
*Solyc06g068600.2*	brown	0.9538	3.68 × 10^−16^	ABC transporter I family member 17
*Solyc12g014290.1*	brown	0.9532	4.44 × 10^−16^	Multiprotein-bridging factor 1b, Transcription factor MBF1
*Solyc06g008350.2*	brown	0.9522	6.00 × 10^−16^	Serine/threonine-protein kinase 19 isoform X1
*Solyc07g006120.2*	brown	0.9511	8.19 × 10^−16^	Autophagy-related protein 18b isoform X2
*Solyc03g122170.2*	brown	0.9498	1.17 × 10^−15^	Peroxisomal (S)-2-hydroxy-acid oxidase GLO4
*Solyc02g093880.2*	brown	0.9495	1.25 × 10^−15^	Transcription factor GTE8, Transcription factor GTE9
*Solyc02g069740.2*	magenta	0.9921	8.90 × 10^−27^	Lysine-specific demethylase JMJ18
*Solyc05g056080.2*	magenta	0.9920	9.81 × 10^−27^	Uncharacterized protein LOC101253087
*Solyc03g115610.2*	magenta	0.9916	2.17 × 10^−26^	Probable LRR receptor-like serine/threonine-protein kinase At1g74360
*Solyc05g008310.2*	magenta	0.9887	1.32 × 10^−24^	G-type lectin S-receptor-like serine/threonine-protein kinase At4g27290, Serine/threonine-protein kinase
*Solyc04g005160.1*	magenta	0.9864	1.67 × 10^−23^	6-phosphogluconate dehydrogenase, decarboxylating 3-like
*Solyc09g010780.2*	magenta	0.9857	3.46 × 10^−23^	Probable protein phosphatase 2C 23, Protein phosphatase 2C/pyruvate dehydrogenase (lipoamide) phosphatase
*Solyc05g055080.1*	magenta	0.9838	1.87 × 10^−22^	Hypothetical protein A4A49_29869
*Solyc02g090970.1*	magenta	0.9817	1.02 × 10^−21^	Mitogen-activated protein kinase kinase kinase NPK1-like, MEKK and related serine/threonine protein kinases
*Solyc10g005630.2*	magenta	0.9816	1.11 × 10^−21^	Putative serine/threonine-protein kinase, Serine/threonine protein kinase
*Solyc10g080010.1*	magenta	0.9808	2.08 × 10^−21^	EGF domain-specific O-linked N-acetylglucosamine transferase

## Data Availability

The raw RNA-seq data had been successfully submitted to the SRA database (PRJNA1045810).

## Supplementary Materials

The following supporting information can be downloaded at: https://www.mdpi.com/article/10.3390/ijms25042187/s1.

## Abbreviations

PYR/PYL: pyrabactin resistance/pyrabactin resistance-like; PP2Cs: 2C protein phosphatases; PP2Cs: type 2C protein phosphatases; SnRK2: sucrose non-fermenting 1-related protein kinase 2; ABA: abscisic acid; RNA-seq: RNA sequencing; NCED: 9-cis-epoxycarotenoid dioxygenase; ABA2: xanthoxin dehydrogenase; AAO3: abscisic-aldehyde oxidase; CYP707A: cytochrome P450 enzyme ABA 8′-hydroxylase; UPLC: ultra-performance liquid chromatography; MS/MS: tandem mass spectrometry; OPLS-DA: orthogonal partial least squares-discriminant analysis; VIP: variable importance in projection.

## References

[1] Anjum S.A., Ashraf U., Tanveer M., Khan I., Hussain S., Shahzad B., Zohaib A., Abbas F., Saleem M.F., Ali I. (2017). Drought induced changes in growth, osmolyte accumulation and antioxidant metabolism of three maize hybrids. Front. Plant Sci..

[2] Chetelat R.T., Pertuze R.A., Faundez L., Graham E.B., Jones C.M. (2009). Distribution, ecology and reproductive biology of wild tomatoes and related nightshades from the Atacama Desert region of northern Chile. Euphytica.

[3] Foolad M.R., Zhang L.P., Subbiah P. (2003). Genetics of drought tolerance during seed germination in tomato: Inheritance and QTL mapping. Genome.

[4] Jia C.P., Guo B., Wang B.K., Li X., Yang T., Li N., Wang J., Yu Q.H. (2022). The LEA gene family in tomato and its wild relatives: Genome-wide identification, structural characterization, expression profiling, and role of *SlLEA6* in drought stress. BMC Plant Biol..

[5] Nguyen N.T., Shivanna H., Savithramma D.L. (2015). Identification of drought tolerant tomato germplasm based on drought tolerant indices. Mysore J. Agric. Sci..

[6] Bahrami F., Arzani A., Karimi V. (2014). Evaluation of yield-based drought tolerance indices for screening safflower genotypes. Agron. J..

[7] Serba D.D., Yadav R.S. (2016). Genomic tools in pearl millet breeding for drought tolerance: Status and prospects. Front. Plant Sci..

[8] Hirayama T., Shinozaki K. (2010). Research on plant abiotic stress responses in the post-genome era: Past, present and future. Plant J..

[9] Zhu T., Zou L., Li Y., Yao X., Xu F., Deng X., Zhang D., Lin H. (2018). Mitochondrial alternative oxidase-dependent autophagy involved in ethylene-mediated drought tolerance in *Solanum lycopersicum*. Plant Biotechnol. J..

[10] Nie S., Huang S., Wang S., Mao Y., Liu J., Ma R., Wang X. (2019). Enhanced brassinosteroid signaling intensity via *SlBRI1* overexpression negatively regulates drought resistance in a manner opposite of that via exogenous BR application in tomato. Plant Physiol. Bioch..

[11] Raghavendra A.S., Gonugunta V.K., Christmann A., Grill E. (2010). ABA perception and signalling. Trends Plant Sci..

[12] Vishwakarma K., Upadhyay N., Kumar N., Yadav G., Singh J., Mishra R.K., Kumar V., Verma R., Upadhyay R.G., Pandey M. (2017). Abscisic acid signaling and abiotic stress tolerance in plants: A review on current knowledge and future prospects. Front. Plant Sci..

[13] Ma Y., Szostkiewicz I., Korte A., Moes D., Yang Y., Christmann A., Grill E. (2009). Regulators of PP2C phosphatase activity function as abscisic acid sensors. Science.

[14] Park S.Y., Fung P., Nishimura N., Jensen D.R., Fujii H., Zhao Y., Lumba S., Santiago J., Rodrigues A., Chow T.F.F. (2009). Abscisic acid inhibits Type 2C protein phosphatases via the PYR/PYL family of START proteins. Science.

[15] Umezawa T., Nakashima K., Miyakawa T., Kuromori T., Tanokura M., Shinozaki K., Yamaguchi-Shinozaki K. (2010). Molecular basis of the core regulatory network in ABA responses: Sensing, signaling and transport. Plant Cell Physiol..

[16] Zhu J.K. (2016). Abiotic stress signaling and responses in plants. Cell.

[17] Ji K., Kai W., Zhao B., Sun Y., Yuan B., Dai S., Li Q., Chen P., Wang Y., Pei Y. (2014). SlNCED1 and SlCYP707A2: Key genes involved in ABA metabolism during tomato fruit ripening. J. Exp. Bot..

[18] Zhang Z., Cao B., Li N., Chen Z., Xu K. (2019). Comparative transcriptome analysis of the regulation of ABA signaling genes in different rootstock grafted tomato seedlings under drought stress. Environ. Exp. Bot..

[19] Zhu M., Meng X., Cai J., Li G., Dong T., Li Z. (2018). Basic leucine zipper transcription factor SlbZIP1 mediates salt and drought stress tolerance in tomato. BMC Plant Biol..

[20] Ma X., Xia H., Liu Y., Wei H., Zheng X., Song C., Chen L., Liu H., Luo L. (2016). Transcriptomic and metabolomic studies disclose key metabolism pathways contributing to well-maintained photosynthesis under the drought and the consequent drought-tolerance in rice. Front Plant. Sci..

[21] Wang P., Yang C., Chen H., Song C., Zhang X., Wang D. (2017). Transcriptomic basis for drought-resistance in *Brassica napus* L. Sci. Rep..

[22] Egea I., Albaladejo I., Meco V., Morales B., Sevilla A., Bolarin M.C., Flores F.B. (2018). The drought-tolerant Solanum pennellii regulates leaf water loss and induces genes involved in amino acid and ethylene/jasmonate metabolism under dehydration. Sci. Rep..

[23] Jaiswal S., Antala T.J., Mandavia M.K., Chopra M., Jasrotia R.S., Tomar R.S., Kheni J., Angadi U.B., Iquebal M.A., Golakia B.A. (2018). Transcriptomic signature of drought response in pearl millet (*Pennisetum glaucum* L.) and development of web-genomic resources. Sci. Rep..

[24] You J., Zhang Y., Liu A., Li D., Wang X., Dossa K., Zhou R., Yu J., Zhang Y., Wang L. (2019). Transcriptomic and metabolomic profiling of drought-tolerant and susceptible sesame genotypes in response to drought stress. BMC Plant Biol..

[25] Arbona V., Manzi M., Ollas D.-C., Gómez-Cadenas A. (2013). Metabolomics as a tool to investigate abiotic stress tolerance in plants. Int. J. Mol. Sci..

[26] Obata T., Witt S., Lisec J., Palacios-Rojas N., Florez-Sarasa I., Yousfi S., Araus J., Cairns J., Fernie A. (2015). Metabolite profiles of maize leaves in drought, heat, and combined stress field trials reveal the relationship between metabolism and grain yield. Plant Physiol..

[27] Tarazona P., Feussner K., Feussner I. (2015). An enhanced plant lipidomics method based on multiplexed liquid chromatography-mass spectrometry revealsadditional insights into cold- and drought-induced membrane remodeling. Plant J..

[28] Chmielewska K., Rodziewicz P., Swarcewicz B., Sawikowska A., Krajewski P., Marczak L., Ciesiolka D., Kuczynska A., Mikolajczak K., Ogrodowicz P. (2016). Analysis of drought-induced proteomic and metabolomic changes in barley (*Hordeum vulgare* L.) leaves and roots unravels some aspects of biochemical mechanisms involved in drought tolerance. Front. Plant Sci..

[29] Pires M.V., Pereira J.A.A., Medeiros D.B., Daloso D.M., Pham P.A., Barros K.A., Engqvist M.K.M., Florian A., Krahnert I., Maurino V.G. (2016). The influence of alternative pathways of respiration that utilize branched-chain amino acids following water shortage in Arabidopsis. Plant Cell Environ..

[30] Savoi S., Wong D.C.J., Arapitsas P., Miculan M., Bucchetti B., Peterlunger E., Fait A., Mattivi F., Castellarin S.D. (2016). Transcriptome and metabolite profiling reveals that prolonged drought modulates the phenylpropanoid and terpenoid pathway in white grapes (*Vitis vinifera* L.). BMC Plant Biol..

[31] Mutwakil M.Z., Hajrah N.H., Atef A., Edris S., Sabir M.J., Al-Ghamdi A.K., Sabir M.J.S.M., Nelson C., Makki R.M., Ali H.M. (2017). Transcriptomic and metabolic responses of Calotropis procera to salt and drought stress. BMC Plant Biol..

[32] Vital C.E., Giordano A., Soares E.D., Williams T.C.R., Mesquita R.O., Vidigal P.M.P., Lopes A.D., Pacheco T.G., Rogalski M., Ramos H.J.D. (2017). An integrative overview of the molecular and physiological responses of sugarcane under drought conditions. Plant Mol. Biol..

[33] Acevedo R.M., Avicol E.H., Gonzalez S., Salvador A.R., Rivarola M., Paniego N., Paniego N., Nunes-Nesi A., Ruiz O.A., Sansberro P.A. (2019). Transcript and metabolic adjustments triggered by drought in Ilex paraguariensis leaves. Planta.

[34] Asakura H., Taira S., Funaki J., Yamakawa T., Abe K., Asakura T. (2022). Mass spectrometry imaging analysis of metabolic changes in green and red tomato fruits exposed to drought stress. Appl. Sci..

[35] Dong S., Ling J., Song L., Zhao L., Wang Y., Zhao T. (2023). Transcriptomic profiling of tomato leaves identifies novel transcription factors responding to dehydration stress. Int. J. Mol. Sci..

[36] Liu M., Zhao G., Huang X., Pan T., Chen W., Qu M., Ouyang B., Yu M., Shabala S. (2023). Candidate regulators of drought stress in tomato revealed by comparative transcriptomic and proteomic analyses. Front. Plant Sci..

[37] Papadopoulou A., Ainalidou A., Mellidou I., Karamanoli K. (2023). Metabolome and transcriptome reprogramming underlying tomato drought resistance triggered by a *Pseudomonas* strain. Plant Physiol. Bioch..

[38] Pirona R., Frugis G., Locatelli F., Mattana M., Genga A., Baldoni E. (2023). Transcriptomic analysis reveals the gene regulatory networks involved in leaf androot response to osmotic stress in tomato. Front. Plant Sci..

[39] Shi J., Du X. (2023). Transcriptome analysis reveals the regulation of cyclic nucleotide-gated ion channels in response to exogenous abscisic acid and calcium treatment under drought stress in tomato. Front. Genet..

[40] Muthusamy M., Uma S., Backiyarani S., Saraswathi M.S., Chandrasekar A. (2016). Transcriptomic changes of drought-tolerant and sensitive banana cultivars exposed to drought stress. Front. Plant Sci..

[41] Svoboda P., Janska A., Spiwok V., Prasil I.T., Kosova K., Vitamvas P., Ovesná J. (2016). Global scale transcriptional profiling of two contrasting barley genotypes exposed to moderate drought conditions: Contribution of leaves and crowns to water shortage coping strategies. Front. Plant Sci..

[42] Novo E., Parola M. (2008). Redox mechanisms in hepatic chronic wound healing and fibrogenesis. Fibrogenesis Tissue Repair..

[43] Miller G., Suzuki N., Ciftci-Yilmaz S., Mittler R. (2010). Reactive oxygen species homeostasis and signalling during drought and salinity stresses. Plant Cell Environ..

[44] Mittler R. (2016). ROS are good. Trends Plant Sci..

[45] Prashanth S.R., Sadhasivam V., Parida A. (2008). Over expression of cytosolic copper/zinc superoxide dismutase from a mangrove plant Avicennia marina in indica rice var Pusa Basmati-1 confers abiotic stress tolerance. Transgenic Res..

[46] Zhang Z., Zhang Q., Wu J., Zheng X., Zheng S., Sun X., Qiu Q.S., Lu T.G. (2013). Gene knockout study reveals that cytosolic ascorbate peroxidase 2 (OsAPX2) plays a critical role in growth and reproduction in rice under drought, salt and cold stresses. PLoS ONE.

[47] Nianiou-Obeidat I., Madesis P., Kissoudis C., Voulgari G., Chronopoulou E., Tsaftaris A., Labrou N.E. (2017). Plant glutathione transferase-mediated stress tolerance: Functions and biotechnological applications. Plant Cell Rep..

[48] Malefo M.B., Mathibela E.O., Crampton B.G., Makgopa M.E. (2020). Investigating the role of Bowman-Birk serine protease inhibitor in Arabidopsis plants under drought stress. Plant Physiol. Bioch..

[49] Wang W.B., Qiu X.P., Kim H.S., Yang Y.X., Hou D.Y., Liang X. (2020). Molecular cloning and functional characterization of a sweetpotato chloroplast IbDHAR3 gene in response to abiotic stress. Plant Biotechnol. Rep..

[50] Verslues P.E., Sharma S. (2010). Proline metabolism and its implications for plant environment interaction. Arab. Book.

[51] Li Z.G., Duan X.Q., Min X., Zhou Z.H. (2017). Methylglyoxal as a novel signalmolecule induces the salt tolerance of wheat by regulating the glyoxalase system, the antioxidant system, and osmolytes. Protoplasma.

[52] Valivand M., Amooaghaie R. (2020). Sodium hydrosulfide modulates membrane integrity, cation homeostasis, and accumulation of phenolics and osmolytes in zucchini under nickel Stress. J. Plant Growth Regul..

[53] Pratelli R., Pilot G. (2014). Regulation of amino acid metabolic enzymes and transporters in plants. J. Exp. Bot..

[54] Hildebrandt T.M., Nunes Nesi A., Araujo W.L., Braun H.P. (2015). Amino acid catabolism in plants. Mol. Plant..

[55] Hu H., Xiong L. (2014). Genetic engineering and breeding of drought-resistant crops. Annu. Rev. Plant Biol..

[56] Soderman E., Mattsson J., Engstrom P. (1996). The Arabidopsis homeobox gene ATHB-7 is induced by water deficit and by abscisic acid. Plant J..

[57] Fujita M., Fujita Y., Maruyama K., Seki M., Hiratsu K., Ohme-Takagi M., Tran L.S.P., Yamaguchi-Shinozaki K., Shinozaki K. (2004). A dehydration-induced NAC protein, RD26, is involved in a novel ABA-dependent stress-signaling pathway. Plant J..

[58] Yang J., Worley E., Udvardi M. (2014). A NAP-AAO3 regulatory module promotes chlorophyll degradation via ABA biosynthesis in Arabidopsis leaves. Plant Cell.

[59] Lee Y.H., Chun J.Y. (1998). A new homeodomain-leucine zipper gene from Arabidopsis thaliana induced by water stress and abscisic acid treatment. Plant Mol. Biol..

[60] Lee Y.H., Oh H.S., Cheon C.I., Hwang I.T., Kim Y.J., Chun J.Y. (2001). Structure and expression of the Arabidopsis thaliana homeobox gene Athb-12. Biochem. Bioph. Res. Co..

[61] Olsson A.S.B., Engstroem P., Seoderman E. (2004). The homeobox genes ATHB12 and ATHB7 encode potential regulators of growth in response to water deficit in Arabidopsis. Plant Mol. Biol..

[62] Huang D., Wu W., Abrams S.R., Cutler A.J. (2008). The relationship of drought-related gene expression in Arabidopsis thaliana to hormonal and environmental factors. J. Exp. Bot..

[63] Guo Y., Gan S. (2006). AtNAP, a NAC family transcription factor, has an important role in leaf senescence. Plant J..

[64] Zhang K., Gan S.S. (2012). An abscisic acid-AtNAP transcription factor-SAG113 protein phosphatase 2C regulatory chain for controlling dehydration in senescing Arabidopsis leaves. Plant Physiol..

[65] Gaudinier A., Rodriguez-Medina J., Zhang L., Olson A., Liseron-Monfils C., Bagman A.M., Foret J., Abbitt S., Tang M., Li B.H. (2018). Transcriptional regulation of nitrogen-associated metabolism and growth. Nature.

[66] Gaudet P., Livstone M.S., Lewis S.E., Thomas P.D. (2011). Phylogenetic-based propagation of functional annotations within the Gene Ontology consortium. Briefings Bioinform..

[67] Zhuang J., Zhang J., Hou X.L., Wang F., Xiong A.S. (2014). Transcriptomic, proteomic, metabolomic and functional genomic approaches for the study of abiotic stress in vegetable crops. Crit. Rev. Plant Sci..

[68] Liu X.Y. (2016). Mapping of QTLs for Salt and Drought Tolerance in Recombinant Inbred Line Population Derived from Wild Tomato *Solanum pimpinellifolium* during Seedling Stage. Master’s Thesis.

[69] Kim D., Landmead B., Salzberg S.L. (2015). HISAT: A fast spliced aligner with low memory requirements. Nat. Methods.

[70] Trapnell C., Williams B.A., Pertea G., Mortazavi A., Kwan G., van Baren M.J., Salzberg S.L., Wold B.J., Pachter L. (2010). Transcript assembly and quantification by RNA-Seq reveals unannotated transcripts and isoform switching during cell differentiation. Nat. Biotechnol..

[71] Shu J.S., Zhang L.L., Liu Y.M., Li Z.S., Fang Z.Y., Yang L.M., Zhuang M., Zhang Y.Y., Lv H.H. (2018). Normal and abortive buds transcriptomic profiling of broccoli ogu cytoplasmic male sterile line and its maintainer. Int. J. Mol. Sci..

[72] Love M.I., Huber W., Anders S. (2014). Moderated estimation of fold change and dispersion for RNA-Seq data with DESeq2. Genome Biol..

[73] Benjamini Y., Hochberg Y. (1995). Controlling the false discovery rate: A practical and powerful approach to multiple testing. J. R. Stat. Soc. B..

[74] Young M.D., Wakefield M.J., Smyth G.K., Oshlack A. (2010). Gene ontology analysis for RNA-Seq: Accounting for selection bias. Genome Biol..

[75] Mao X., Cai T., Olyarchuk J.G., Wei L. (2005). Automated genome annotation and pathway identification using the KEGG Orthology (KO) as a controlled vocabulary. Bioinformatics.

[76] Wallenius K.T. (1963). Biased sampling: The Non-Central Hypegeometric Probability Distribution. Ph.D. Thesis.

[77] Chen W., Gong L., Guo Z., Wang W.S., Zhang H.Y., Liu X.Q., Yu S.B., Xiong L.Z., Luo J. (2013). A novel integrated method for large-scale detection, identification, and quantification of widely targeted metabolites: Application in the study of rice metabolomics. Mol. Plant.

