# Supplementary material for: Transcriptome Analysis and Metabolic Profiling Reveal the Key Regulatory Pathways in Drought Stress Responses and Recovery in Tomatoes
Source: Int J Mol Sci. 2024 Feb 11;25(4):2187. doi: 10.3390/ijms25042187 (PMC10889177; doi:10.3390/ijms25042187)
Supplement: Supplementary file 1 [file ijms-25-02187-s001.zip › Additional file2 Figures.pdf]

Journal: International Journal of Molecular Sciences

Title: Transcriptome analysis and metabolic profiling reveal the key regulatory pathways in drought stress responses and recovery in tomato

Author names: Jinshuai Shu[1, †, *], Lili Zhang[2, †], Guiming Liu[2], Xiaoxuan Wang[1], Fuzhong Liu[1], Ying Zhang[1], Yuhui Chen[1]

Affiliation: [1]State Key Laboratory of Vegetable Biobreeding, Institute of Vegetables and Flowers, Chinese Academy of Agricultural Sciences, Beijing 100081, China; [2]Beijing Key Laboratory of Agricultural Genetic Resources and Biotechnology, Institute of Biotechnology, Beijing Academy of Agriculture and Forestry Sciences, Beijing, 100097, China

[*]Corresponding author Email: shujinshuai@caas.cn

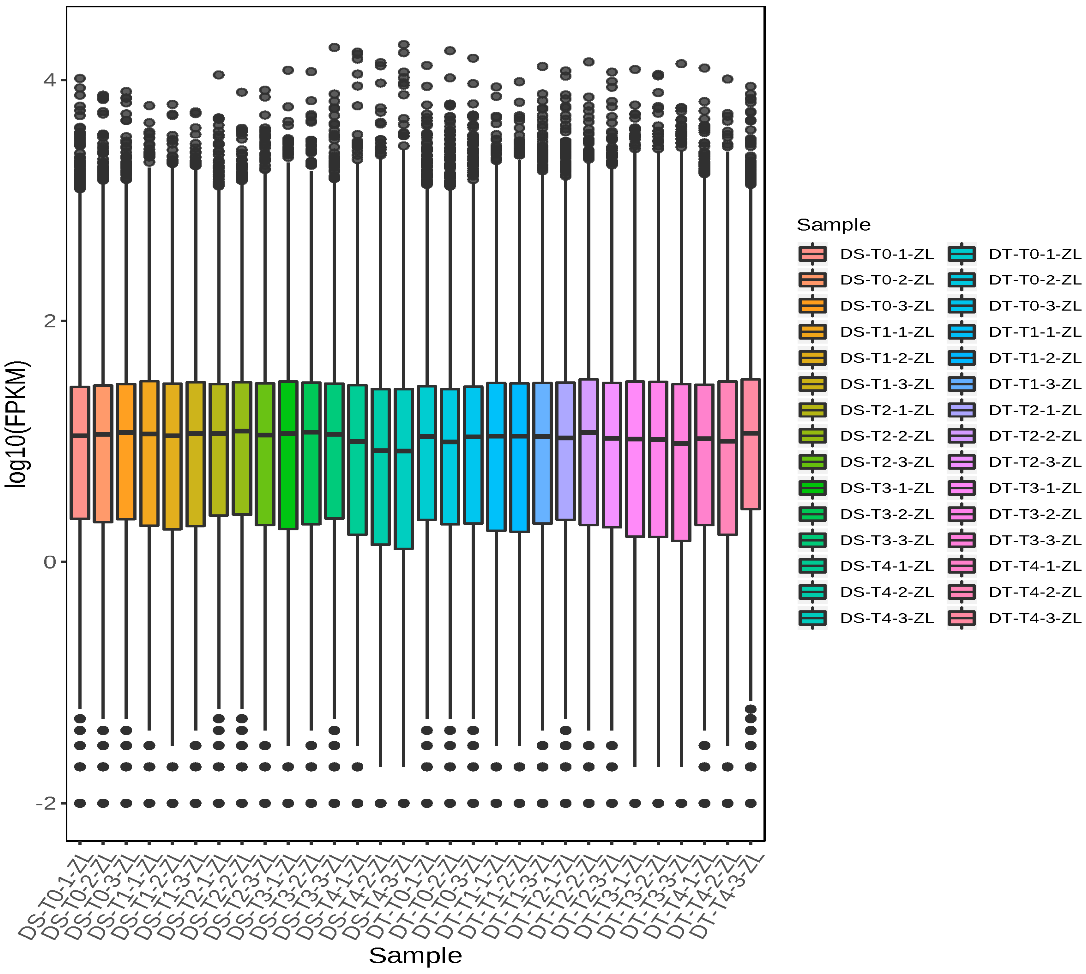

Figure S1 Box plot of RNA-seq samples in DS and DT genotypes

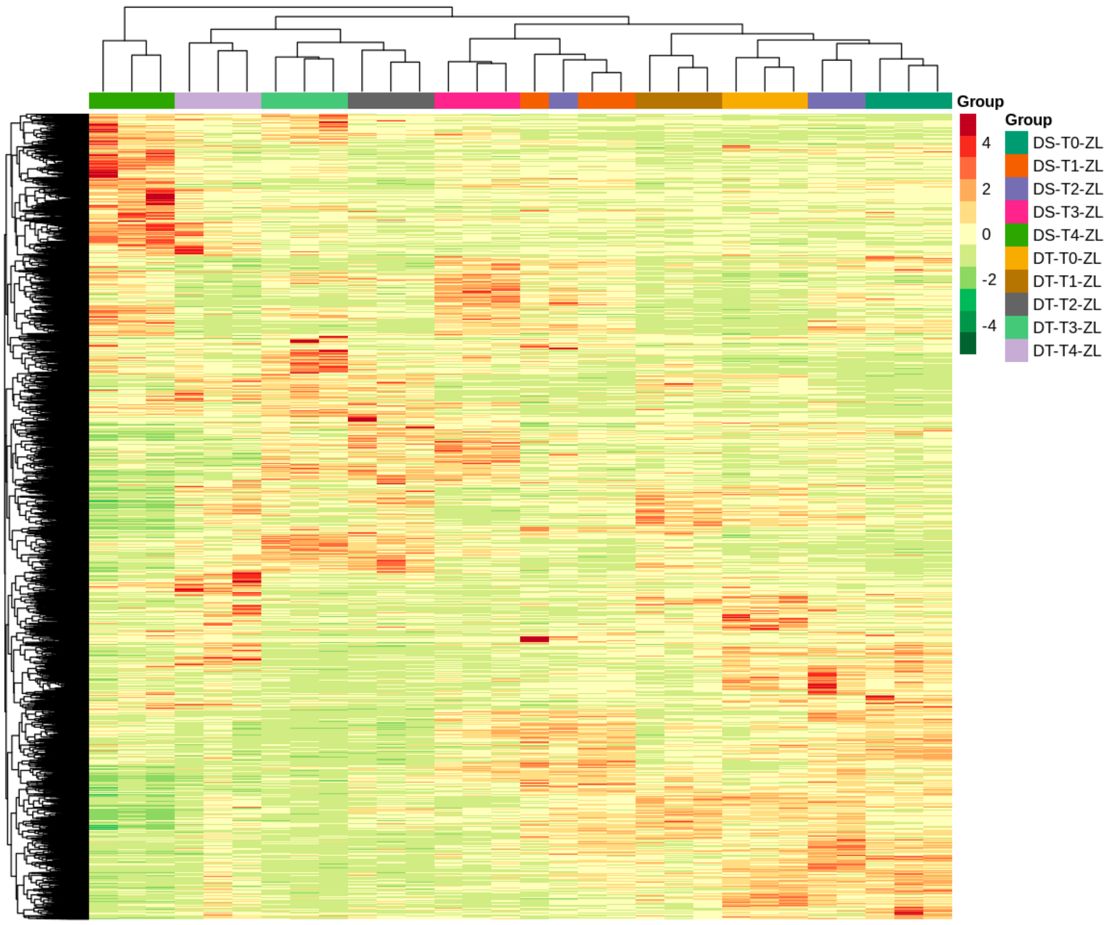

Figure S2 Cluster heat map of RNA-seq samples in DS and DT genotypes

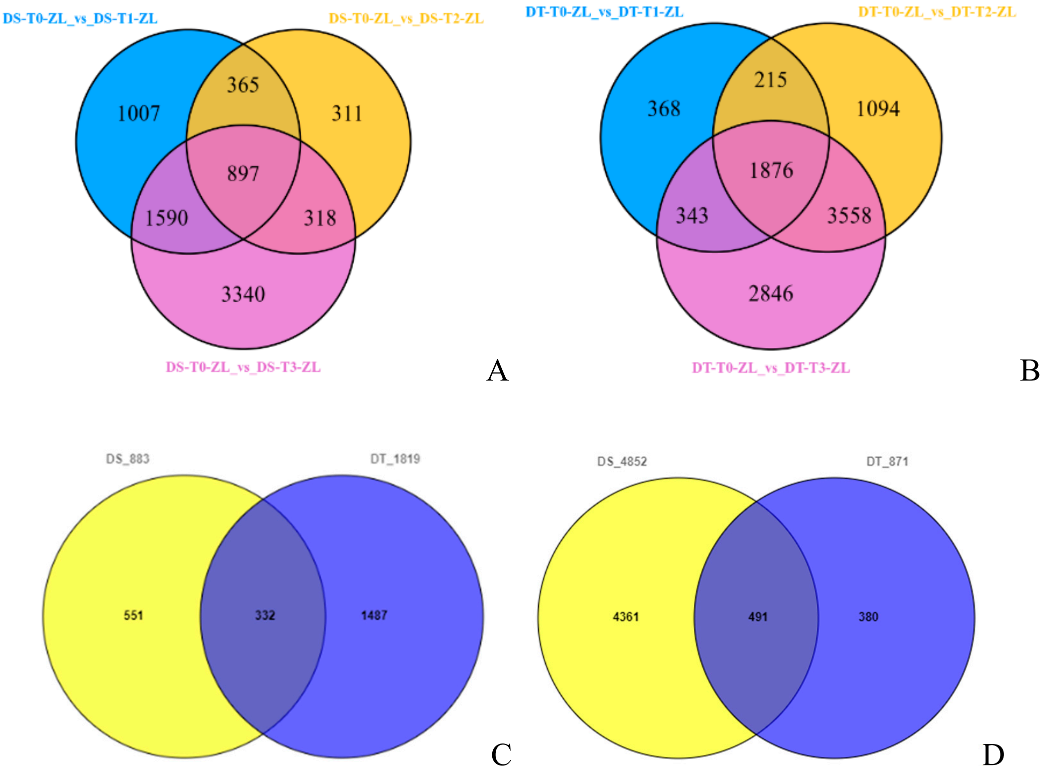

Figure S3 Venn diagrams of DEGs common to DS and DT under drought and recovery conditions. A: Venn diagram of DEGs in DS. B: Venn diagram of DEGs in DT. C: Venn diagram of the genes identified as DEGs at all drought stress time-points in DS and DT. D: Venn diagram of the genes identified as DEGs between DS and DT during rehydration.

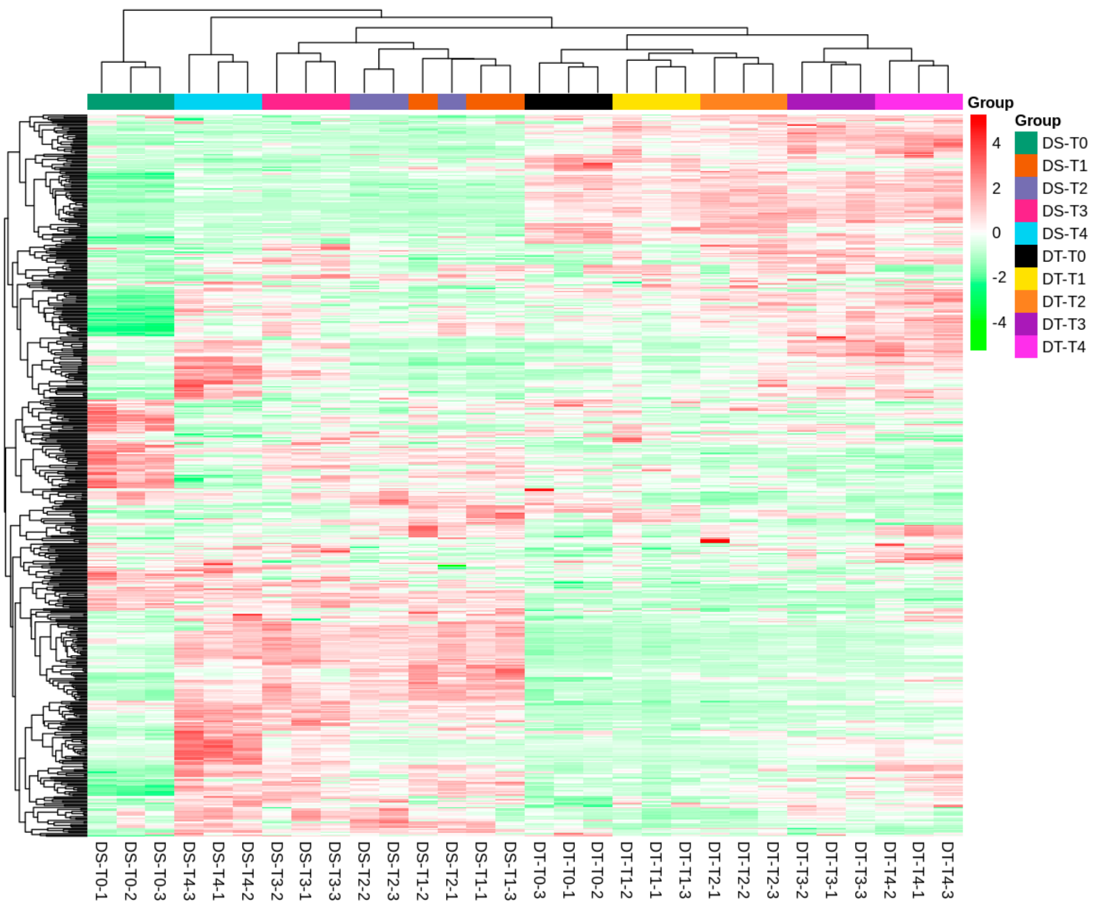

Figure S4 Correlations among 30 metabolome samples in the DS and DT genotypes.

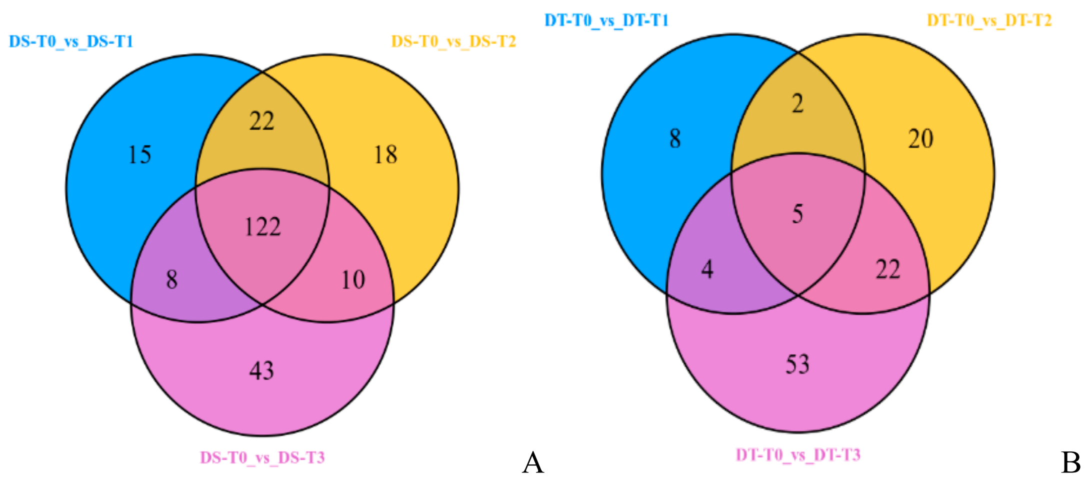

Figure S5 Venn diagram of the differentially abundant metabolites in DS and DT under drought conditions. A: Venn diagram of differentially abundant metabolites in DS. B: Venn diagram of differentially abundant metabolites in DT.
